# Structured Light Camera’s Point Clouds Captured and Stitched by Humanoid for 3D Objects Based on ICP Registration Algorithm

**DOI:** 10.3390/biomimetics11070449

**Published:** 2026-06-29

**Authors:** Hong-Yu Lin, Che-Ping Hung, Kuo-Yang Tu, Fang-Tsen Kuo

**Affiliations:** 1 Ph.D. Program in Engineering Science and Technology, College of Engineering, National Kaohsiung University of Science and Technology, No. 1, University Road, Yanchao District, Kaohsiung City 824, Taiwan; 2 Department of Electrical Engineering, National Kaohsiung University of Science and Technology, No. 1, University Road, Yanchao District, Kaohsiung City 824, Taiwan

**Keywords:** humanoid, structured light, point cloud registration, 3D reconstruction, ICP algorithm, automated alignment

## Abstract

In recent decades, humanoids have become more popular in various applications. However, their applications in human life are more than those in industry. In this paper, a humanoid is used to capture the sets of point clouds of an object for three-dimensional reconstruction. The structured light camera is widely used across diverse 3D scanning applications due to its high resolution, rapid acquisition capability, and adaptability to various material surfaces. Therefore, the humanoid developed by our team holds a structured light camera which captures the point clouds of an object put on a platform for the reconstruction of its 3D digital model. The platform is rotated so that the structured light camera can capture the image of all view angles on the object. Meanwhile, the structured light camera captures point clouds, and the camera of the humanoid recognizes the QR code on the platform so that the sets of point clouds can be distinguished by view angles on the object. Then, the automated registration process of the point cloud sets for a 3D model based on the point-to-plane iterative closest point (ICP) algorithm is proposed. The process incorporates preprocessing techniques, such as downsampling and normal vector estimated from plane, and utilizes the ICP algorithm for registration, ultimately achieving markerless and precision automatic merging of multi-view point cloud data. Experimental results demonstrate that the proposed method with the humanoid can effectively improve the completeness and accuracy of 3D reconstruction models, significantly reduce manual intervention, and enhance the system’s versatility and practical feasibility. Key parameters adjusted for more efficient computation of the ICP algorithm are revealed. In addition, the experimental results of the proposed ICP compared with G-ICP are also included.

## 1. Introduction

In recent decades, humanoids have become more popular in various applications. However, their applications in human life are more than those in industry. The humanoids are usually designed to possess capabilities that are similar to those of humans. The full capability of humanoids needs to be extended for industrial scenarios such as use on a manufacturing line, factory, etc. In this paper, a humanoid is used to capture the sets of a point cloud of an object for three-dimensional information reconstruction.

With the rapid advancement of three-dimensional sensing technologies and the growing demand for digital modeling, optical non-contact measurement has been widely adopted in precision manufacturing, industrial inspection, virtual exhibition, and cultural heritage preservation [1]. Among these technologies, the image captured by a structured light camera [2] offers notable advantages for short-range scanning due to its high spatial resolution, fast acquisition, and strong adaptability to surfaces with diverse material properties and has therefore been extensively applied.

The fundamental principle of structured light imaging is to project a predefined light pattern, such as dots, stripes, or coded patterns, onto an object surface and capture the resulting deformation of the projected pattern using a camera [3,4]. Depth information corresponding to each pixel can then be estimated via triangulation and subsequently converted into a dense point cloud, which serves as the basis for downstream 3D modeling and geometric analysis [5,6]. However, point clouds captured from a single viewpoint are inherently affected by occlusions and viewing-angle limitations, preventing complete reconstruction of an object’s overall geometry. Consequently, point clouds acquired from multiple viewpoints must be aligned within a common coordinate system and stitched together to form a complete 3D geometric model [7,8].

Traditionally, the stitching of multi-view point clouds often requires manual assistance, such as markers, calibration spheres, or other geometric references, to facilitate initial alignment and subsequent fine registration. In addition, some approaches rely on external sensing devices such as optical tracking systems [9], rotary encoders [10], or inertial navigation units [11] with pose estimation capability to improve registration accuracy and robustness. Although these methods can perform well in certain scenarios, they frequently depend on hardware setup, operator expertise, and environmental conditions, which limits system flexibility and portability [12,13]. Such requirements are also unsuitable for applications where the target object cannot be altered or interfered with, including cultural artifact digitization and in situ modeling. Therefore, developing an automated technique of stitching point clouds that performs feature correspondence and geometric refinement without manual markers has become an important research trend in 3D scanning and reconstruction.

In light of these challenges, it is valuable to propose a highly autonomous method and system of stitching 3D point clouds that can efficiently fuse multi-view point clouds without manual intervention, marker placement, or external auxiliary devices. Such a strategy improves general applicability, operational flexibility, and feasibility for on-site deployment.

Research on 3D imaging has expanded rapidly, accelerating technology transfer into a wide range of practical applications. Various sensing technologies have been developed for acquiring 3D point clouds, including structured light systems, time-of-flight (ToF) sensors, phase-difference measurement systems, and laser scanning techniques [14]. These technologies are different from measurement principles, sensing range, spatial resolution, and noise characteristics, which may lead to variations in the quality of the captured point cloud data and may further influence the performance of subsequent processing tasks such as the 3D registration and reconstruction of point clouds. It is notable that structured light is particularly suitable for near-range, high-resolution applications [14]. In optical 3D imaging, structured light systems are widely recognized for their superior scanning accuracy and stability [15]. Geng [2] analyzed structured light imaging principles and applications from the perspectives of projection patterns and system architectures. Regarding coding strategies, Salvi et al. [3] surveyed and compared spatial coding, temporal coding, and direct coding methods, discussing their applicable scenarios and accuracy characteristics. Pages et al. [16] further proposed a De Bruijn sequence-based stripe coding scheme to enhance pattern recognition stability and robustness against interference. In domestic studies, Chou investigated an octal tic-tac-toe coding design to improve localization accuracy [17], while Lau provided a comprehensive application-oriented overview of structured light technology [18].

For the stitching and registration of point clouds, the iterative closest point (ICP) algorithm proposed by Besl and McKay has become a classical method [19]. ICP aligns point clouds by iteratively searching for nearest neighbors and estimating rigid transformation, and it has been widely used in 3D reconstruction and scan data integration. To address ICP’s sensitivity to initial alignment and its susceptibility to noise, Segal et al. proposed generalized ICP (G-ICP) [20], which combines point-to-point and point-to-plane models and introduces Gaussian-based error modeling to improve stability and accuracy. In addition, the algorithmic taxonomy and performance analysis of stereo correspondence by Scharstein and Szeliski provides useful references for disparity estimation and structured light pattern matching [21].

Regarding feature descriptors and registration methods, Huang et al.’s Predator introduced an overlapping region prediction mechanism for the first time, significantly improving registration performance in low-overlap scenarios [22]. The GeoTransformer proposed by Qin et al. adopts a geometric transformer architecture, encoding point-to-point distance and triangular angle information to achieve invariance to rigid body transformations, achieving high-precision registration without RANSAC [23]. Yu et al. proposed RoITr to design rotation-invariant transformers, further improving the robustness of geometric descriptors to rotational changes [24]. The BUFFER proposed by Ao et al. balances registration accuracy, computational efficiency, and generalization ability with only 0.92 M parameters [25]. In terms of a literature review, Zhang et al. proposed a complete classification system covering supervised and unsupervised methods and systematically compared more than 100 registration methods under a unified experimental setting [26]. In the field of deep learning registration methods, Wang et al. utilized an attention mechanism to predict soft correspondences between point clouds and combine differentiable SVD to estimate rigid body transformations, representing a representative work in early end-to-end deep learning registration [27]. RPMNet learns soft correspondences from mixed features through differentiable sinkhorn layers, demonstrating strong adaptability to partially visible point clouds [28]. PointNetLK Revisited improves upon the original PointNetLK by introducing an analytical Jacobian matrix to enhance numerical stability and generalization ability; it also improves global registration [29].

In practical 3D scanning, a set of point clouds captured from a single viewpoint typically covers only a portion of an object surface and is insufficient for full reconstruction of the global geometry. Therefore, to obtain complete shape information, multiple sets of point clouds must be captured from different positions and viewing angles and then integrated. This integration process is commonly referred to as point cloud registration, whose core objective is to align point clouds from different coordinate systems into a unified reference frame through spatial transformations, thereby constructing a consistent and complete 3D model.

Extending the application scenarios of humanoids has been an extremely hot research topic in recent years. Some researchers focus on arms and hands. The dexterous hand plays crucial roles in the process of humanoid execution. S. Cao et al. studied multi-finger synergistic movement for the dexterous hand of humanoids [30]. Dual-arm humanoids offer the advantages of higher load capacity, improved operational efficiency, etc. Thus, F. Ju et al. proposed a dual-arm reactive synchronized motion controller (SMC) by incorporating closed-chain constraints [31]. Multiple humanoid robot manipulators in industrial and collaborative settings remain a significant challenge. M. Özbaltan et al. proposed a framework for modeling task scheduling for multiple humanoid robot manipulators by using the symbolic discrete controller synthesis technique [32]. In this paper, the combined use of a humanoid’s structured light camera and a camera for the reconstruction of its 3D digital model is proposed. A structured light camera captures the point clouds of an object, and the humanoid camera recognizes the view angle. Thus, the point clouds combined with the view angles can be stitched together for a 3D digital model of the object. After getting the multi-view point clouds, the ICP algorithm is proposed to stitch the captured point clouds for the 3D object model.

The common methods of point cloud registration can be categorized into three major types:

1. Geometric registration: directly aligns point clouds using geometric point coordinates; a representative method is ICP.
2. Feature-based registration: extracts distinctive local or global descriptors from point clouds (e.g., FPFH, SHOT) for matching and alignment.
3. Image-assisted registration: incorporates color image information to support point cloud alignment, often used in RGB-D camera applications.

Based on the above research background and technical challenges, in this paper, an automated 3D modeling pipeline based on structured light imaging is developed. First, multi-view point cloud data are acquired using a structured light sensor and preprocessed to remove noise. Normal vectors are then estimated, and multi-view point cloud stitching is performed using the ICP algorithm. Finally, the integrated point cloud is used to reconstruct a complete 3D model of the target object. The proposed method can automatically complete point cloud alignment and reconstruction without manual markers or external auxiliary devices, improving the automation level, accuracy, and application flexibility of 3D modeling. It provides practical contributions and application value for digital manufacturing, cultural heritage preservation, and on-site scanning.

The remaining sections of this paper are organized as follows. The problem is addressed in this work and major challenges encountered in current 3D modeling are discussed in Section 2. Section 3 describes the proposed solutions and the overall methodology. Section 4 present the experimental design and the experimental results, respectively, including quantitative data and visual demonstrations. Section 5 concludes this research and outlines directions for future improvements and extensions.

## 2. Problem Statements

During 3D scanning and modeling, reconstructing an object’s complete geometric shape requires aligning and stitching multiple point clouds acquired from different viewpoints. However, point clouds captured from a single view are often constrained by viewpoint limitations and occlusions, causing certain regions to be incompletely sampled. As a result, the reconstructed model may exhibit structural gaps or surface discontinuities. In addition, optical measurement is highly sensitive to ambient illumination, object material properties, and surface reflectance. When the target surface is highly reflective or has low contrast, depth information can be partially lost and the point cloud may become sparse, which further degrades the overall modeling quality.

During multi-view integration, point cloud datasets often suffer from scale discrepancies, pose deviations, or insufficient overlap between views. Point clouds captured from different viewpoints may exhibit geometric inconsistencies due to variations in viewing angle or sensor-related errors, which can lead to translation and rotation misalignments or boundary discontinuities in the stitched model. This issue becomes more pronounced for curved or symmetric objects, where the absence of salient geometric features increases the likelihood of incorrect correspondences and results in unstable registration. Moreover, noise and outliers in the raw data can interfere with normal vector estimation and hinder the convergence of the ICP algorithm, thereby reducing stitching accuracy.

Traditionally, the stitching of multi-view point clouds often requires manual assistance such as placing fiducial markers, calibration spheres, or reference objects with known geometries to establish an initial alignment and enable subsequent fine registration [32]. Another class of approaches relies on external sensing devices, including optical tracking systems, rotary encoders, or inertial navigation units, to improve registration accuracy and robustness. Although these methods can effectively enhance precision in certain applications, they are highly dependent on hardware setup, operator proficiency, and environmental conditions. This reliance reduces portability and practical flexibility, and it is unsuitable for scenarios such as cultural heritage digitization and on-site modeling, where markers cannot be attached to the object or additional devices cannot be installed.

In summary, the 3D modeling pipeline faces multiple challenges, including sensor noise, data loss caused by occlusions and blind viewing angles, insufficient features that lead to incorrect matching, and the strong sensitivity of registration algorithms to initialization and data quality. Achieving accurate and automated alignment and stitching of multi-view point clouds without relying on manual markers or external positioning devices while simultaneously maintaining both modeling efficiency and reconstruction accuracy has become a critical research problem in contemporary 3D scanning and reconstruction.

To address the aforementioned issues, in this paper, the humanoid holding a structured light camera for 3D digital modeling of objects is proposed. The object is installed on a rotating platform. When the structured light camera captures the point clouds of the object, the humanoid camera reads the QR code on the rotating platform to recognize the view angle. Thus, the humanoid can capture the sets of point clouds and their corresponding view angles. Among the major 3D vision sensing technologies, structured light imaging is characterized by non-contact measurement, high spatial resolution, and high accuracy and has become an important option in industrial automation and machine vision applications. Its core principle is based on triangulation, in which a projector and a camera with a known geometric relationship form the fundamental measurement setup.

During measurement, the projector casts a predesigned optical pattern onto the target surface. When the pattern illuminates a 3D surface with height variations, the observed pattern exhibits geometric deformation driven by depth changes. The camera, positioned at a different viewpoint, captures the deformed pattern and quantifies the pattern displacement in the image. By integrating the geometric relationship between the projector and the camera, including extrinsic parameters, with the camera imaging model and intrinsic parameters, the system solves the inverse triangulation geometry to estimate the 3D coordinates of surface points. In this way, a point cloud representation of the target object can be obtained.

To improve the accuracy and stability of 3D reconstruction, most structured light systems adopt stripe encoding patterns, among which a hybrid scheme combining a Gray code and phase-shifting stripes is the most common. Gray code patterns project a sequence of black and white images with a bit transition logic, enabling each pixel in the captured images to be assigned a unique stripe code while reducing decoding errors. Phase shifting projects sinusoidal stripe patterns with multiple phase offsets, allowing the system to estimate the phase information from pixel intensity variations and thereby achieve subpixel depth resolution. By combining these two approaches, the system can effectively mitigate the limitations of conventional encoding methods in terms of spatial resolution and code uniqueness and further improve the overall quality of 3D reconstruction.

In this study, the Mech-Eye PRO S structured light camera was selected. The device is developed by Mech-Mind Robotics and is designed for static 3D scanning in industrial environments. It adopts a classical configuration consisting of one projector and one camera together with an integrated high-resolution imaging module and a precision optical projection unit. The system can stably project multiple sets of stripe and phase patterns, and it performs rapid multi-frame acquisition and processing through synchronized control.

The Mech-Eye PRO S includes built-in encoding and decoding algorithms and a triangulation module, enabling dense 3D data reconstruction and output within a very short time. The output is provided as point cloud data, which is a collection of 3D spatial coordinates corresponding to surface points of the object, including X, Y, and Z values. Additional attributes such as grayscale intensity, color texture (RGB), and surface normals can also be included. This point cloud format can be directly used in a wide range of applications, including dimensional measurement, shape comparison, CAD alignment, and robot guidance.

The Mech-Eye PRO S can reliably acquire depth information from highly reflective, dark-colored, or texture-rich objects. Its algorithms also provide strong robustness to ambient light interference and include an automatic exposure control mechanism, enabling effective adaptation to dynamic industrial conditions. Overall, the Mech-Eye PRO S integrates high-quality optical components, mature stripe encoding techniques, and enhanced point cloud output capability, making it an ideal choice for the good-resolution 3D reconstruction tasks in this study. The generated point cloud data will serve as the primary input for subsequent geometric analysis and algorithm development, supporting the construction of an accurate and reliable 3D visual perception module.

In this paper, an automated 3D modeling approach that integrates structured light imaging with a multi-stage point cloud processing pipeline to construct complete geometric models is proposed. The proposed workflow covers point cloud preprocessing, normal vector estimation, density control, initial alignment, and fine registration, with the goal of improving stitching accuracy and model completeness. The overall system emphasizes automation, robustness, and general applicability, aiming to achieve efficient and good precision multi-view point cloud integration and 3D modeling without manual intervention or external auxiliary devices.

## 3. Point Cloud Registration

The procedure of point cloud registration can be divided into two stages: coarse registration and fine registration. In the coarse registration stage, an initial rigid transformation, including rotation and translation, is estimated between the source and target sets of the point cloud. The source set of the point cloud refers to the dataset that will undergo spatial transformation, whereas the target set of the point cloud serves as the fixed reference. This step brings their overlapping regions into approximate alignment and provides a favorable initialization for the subsequent stage of fine registration.

After the initial alignment, the system proceeds to fine registration to refine the alignment results and improve registration accuracy. In this study, a geometric registration approach is adopted in the stage of fine registration, with the classical ICP algorithm serving as the core method. ICP repeatedly establishes correspondences between point pairs and incrementally estimates the rigid transformation to minimize the error of objective function. As a result, good accuracy alignment of the point cloud can be achieved, providing a solid foundation for the subsequent tasks of 3D reconstruction.

A structured light camera captures point cloud data of 3D objects. To improve both accuracy and computational efficiency of point clouds for applications such as 3D modeling, registration, and geometric analysis, the preprocessing stage plays a critical role. Through systematic data cleaning and simplification, preprocessing can effectively reduce noise and redundant information while preserving important geometric features. In general, the preprocessing operations of point clouds include downsampling, denoising and filtering, normal vector estimation, plane removal, coordinate transformation, and cropping. In this study, normal vector estimation and downsampling are adopted as the main preprocessing steps, followed by point cloud stitching using the ICP method.

### 3.1. Normal Vector Estimation

In 3D processing, point cloud data only consist of discrete spatial coordinates and therefore lack explicit topology and surface information. As a result, local geometric properties are often inferred by analyzing the structure of a point’s neighborhood. The normal vectors of a surface represent a critical property and are widely used in many processing tasks of 3D geometry, including surface reconstruction, edge detection, feature descriptor generation, and point cloud registration. The primary goal of normal vector estimation is to assign each point a unit vector that is perpendicular to the local surface represented by its neighborhood. This vector characterizes the differential geometric direction at that point and facilitates further analysis, as well as reconstruction of the overall spatial structure.

Let the set of an object point cloud captured by structured light camera be denoted as $P=\{p_{i}\;,\;i=1\ldots n\}$, where each point $p_{i}\in R^{3}$ and n is the total number of points. In the geometric analysis of the point cloud, a local neighborhood must be defined for each point. For any point p, a set of neighboring points is collected from the surrounding space to form a set of neighborhood points. Let the neighborhood of p be denoted as $N(p)=\{p_{i}\;,\;i=1\ldots k\}$, where k is the number of neighboring points. By analyzing N(p), the local geometric properties of the region can be characterized.

This neighborhood set provides the essential basis for subsequent geometric computations, including normal vector estimation, curvature analysis, and feature descriptor extraction. A common approach is the k-nearest neighbors (KNN) algorithm. Specifically, given a query point p, the neighborhood N(p) is constructed by selecting the k points with the smallest Euclidean distances to p. An advantage of this method is that each neighborhood contains a fixed number of points, which helps mitigate the impact of non-uniform point cloud density on the quality of normal vector estimation and other geometric feature computations.

After obtaining the neighborhood point set *N*(*p*), an optimal plane can be estimated to characterize the local geometry. First, the geometric centroid of *N*(*p*) is computed. Next, the covariance matrix is constructed to capture the principal directions of the local point distribution, and a local plane is fitted accordingly. This procedure is commonly formulated as a least squares problem, where the plane is estimated by minimizing the sum of squared perpendicular distances from the points in *N*(*p*) to the plane. The resulting plane represents the local tangent plane of the surface around, *p* providing a geometric basis for subsequently deriving the normal vectors and curvature of surface.

The geometric centroid C of *N*(*p*) is computed as follows:

(1) $$c=\frac{1}{k}\sum_{i=1}^{k}p_{i}$$

Let the offset vector of each neighborhood point relative to the centroid be denoted as $d_{i}=p_{i}-c$. Using these offsets, the 3D covariance matrix can be constructed as follows:

(2) $$C=\frac{1}{k}\sum_{i=1}^{k}d_{i}{d_{i}}^{T}$$

where ${d_{i}}^{T}$ denotes the transpose of the vector $d_{i}$. Since $d_{i}{d_{i}}^{T}$ is an outer product, the resulting covariance matrix C is a unique symmetric positive semidefinite matrix, whose elements reflect the variance and correlation among the coordinate components.

Next, an eigenvalue decomposition is performed on C as follows:

(3) $$Cv_{j}=\lambda_{j}v_{j}\;,\;\;\;j=1,2,3$$

where $\lambda_{j}$ and $v_{j}$ denote the j-th eigenvalue and its corresponding eigenvector, respectively. Since C is a real symmetric matrix, its three eigenvectors are mutually orthogonal. The associated eigenvalues quantify the variance of the neighborhood point distribution along the three principal directions.

Let the smallest eigenvalue be denoted as $\lambda_{min}=\underset{j}{\min}\lambda_{j}$. The eigenvector corresponding to $\lambda_{min}$ is denoted as $v_{min}$. The vector $v_{min}$ represents the normal direction of the locally approximated plane fitted to the neighborhood point set. Using this approach, the surface normal of the locally approximated plane can be estimated for each neighborhood, providing geometric information that supports the registration of the point cloud for 3D reconstruction.

After eigenvalue decomposition, the eigenvector $v_{min}$ associated with the smallest eigenvalue $\lambda_{min}$ indicates the direction along which the set of neighborhood points exhibits the least variation. Therefore, it can be regarded as the normal direction of the locally fitted plane. In other words, this direction is orthogonal to the dominant distribution plane of the neighborhood points and can effectively characterize the local geometric property at the point.

However, point cloud data does not inherently encode orientation, and the estimated normal vector may have two opposite but equally valid directions. To ensure consistent normal vector orientation, this study makes use of the sensor viewpoint as a reference and enforces a unified normal direction, thereby preventing geometric discontinuities or incorrect correspondences in subsequent registration and reconstruction processes.

The quality of normal vector estimation is influenced by multiple factors, among which the major critical parameter is the neighborhood size, that is, the number of neighboring points k. When k is too large, noise can be effectively smoothed, and the estimated normal vector tends to be more stable. However, local geometric details may be excessively smoothed, which can blur shape boundaries and lead to feature loss. In contrast, when k is too small, more fine-scale details can be preserved, but the estimation becomes highly sensitive to noise, often resulting in abrupt changes in normal directions and reduced stability.

In addition, noise in the raw data can significantly degrade the accuracy of local plane fitting. In such cases, smoothing is often required, for example by using the mean least squares (MLS) method, to improve estimation stability and noise robustness. Another important factor is non-uniform point density. In regions with large density variations, a fixed neighborhood parameter may lead to overfitting or underfitting. Therefore, an adaptive neighborhood selection strategy can be employed to accommodate the point distribution characteristics in different regions.

Finally, when the raw data of a point cloud is overly dense, the normal vectors of the surface may become discontinuous or oscillatory across adjacent regions, which can compromise the stability of subsequent registration and reconstruction. Appropriate downsampling can effectively alleviate this issue. It not only balances estimation stability and detail preservation but also improves overall computational efficiency. In summary, normal vector estimation should be configured according to the characteristics of the point cloud data and the requirements of the target application. Key factors, such as neighborhood size, smoothing strategy, and density adjustment, should be considered jointly in order to obtain an estimated normal vector that is accurate and stable.

### 3.2. Downsampling

During point cloud registration, raw data that is excessively dense can substantially increase computational cost and may lead to issues such as unstable normal vector estimation, redundant features, and inefficient data storage. These influences not only degrade the accuracy and robustness of registration results but also significantly prolong the overall processing time. To improve computational efficiency and reduce data redundancy, downsampling has become an essential step in the preprocessing stage of point cloud registration.

The purpose of downsampling is to reduce the numbers in a set of point clouds while preserving their geometric structure and dominant features, thereby improving the computational efficiency and robustness of subsequent algorithms. In this paper, voxel downsampling is adopted as the primary reduction strategy. This method is an optimization approach based on spatial partitioning and is widely used in the preprocessing of point clouds for 3D reconstruction. Its core idea is to divide the 3D space into uniformly sized cubic grids with a specified voxel edge length ν, along the x, y, and z axes. Let $p_{min}$ denote the minimum boundary point of the point cloud in 3D space, that is, the minimum values across all dimensions of the point set. It is defined as follows:

(4) $$p_{min}={[min}_{i=1}^{m}p_{i}^{x},\;{\;min}_{i=1}^{m}p_{i}^{y},\;{\;min}_{i=1}^{m}p_{i}^{z}{]}^{T}$$

where m is the total numbers in the set of point cloud P.

The geometric centroid of all points within the same voxel is computed and used as the representative point of that voxel. This approach effectively reduces data size while preserving the object’s geometric outline and spatial distribution characteristics, thereby simplifying the dataset and accelerating subsequent algorithms. The formulation is given as follows:

(5) $$V\left(p_{i}\right)=\left\lfloor \frac{p_{i}-p_{min}}{\nu }\right\rfloor$$

where ⌊•⌋ denotes the floor operator; i. e. round down to an integer, $p_{i}$ is the 3D coordinate of the i -th point in the set of point cloud, that is, $p_{i}=(p_{i}^{x},p_{i}^{y},p_{i}^{z})$; and ν is the edge length of voxel.

After voxel downsampling, the total number of points m in the point cloud P is reduced, which improves computational efficiency and yields a simplified structure for an easier process. By using spatial indexing or a hash table, voxel indices can be constructed efficiently, enabling effective handling of large-scale data of point clouds. This method provides good stability in predictable behavior. Moreover, the voxel edge length ν can be adjusted to precisely control the data size and sampling density after downsampling, achieving a practical balance between computational speed and geometric fidelity. Therefore, voxel downsampling is well suited for the initial reduction of large point clouds to shorten subsequent registration time.

However, voxel downsampling has inherent limitations. When the voxel edge length is set too large, fine details and boundary information can be overly smoothed, making it difficult to preserve high-frequency geometric structures. In contrast, if the voxel size is too small, the reduction in data volume becomes limited, weakening the overall downsampling effect. In addition, this method is a non-feature-aware downsampling strategy, meaning it does not adaptively select points based on geometric characteristics such as curvature variations or edge contours. As a result, important structural information may be lost. Furthermore, the discretized voxel boundaries can intersect object edges, which may blur or smooth local contours and subsequently affect the accuracy of later reconstruction and matching. As a result, the size of voxel downsampling is a trade-off parameter between computation time and performance. Normally, it is adjusted for the objects in different surface features.

Overall, voxel downsampling offers advantages such as fast computation, a simple and structured representation, and high flexibility in controlling the sampling density. Nevertheless, the voxel size must be selected by balancing the target application requirements and the characteristics of the input data. With an appropriate voxel resolution, key geometric features can be preserved while significantly improving the efficiency and stability of subsequent normal vector estimation and registration.

### 3.3. Iterative Closest Point

The iterative closest point (ICP) algorithm, proposed by Besl and McKay in 1992 [7], is one of the most widely used methods for point cloud registration. Its objective is to accurately align a source set of point clouds to a target set of point clouds. ICP is particularly effective when an approximate initial alignment between the two sets of point clouds is already available.

ICP iteratively refines the spatial pose of the source set of point clouds to minimize the geometric discrepancy between the source and target sets of point clouds. Its procedure can be summarized as follows. First, an initial relative pose between the source and target point clouds is provided. Next, for each point in the source point cloud, the nearest neighbor in the target set of point clouds is searched to establish point correspondences. Based on these matched pairs, a rigid transformation, consisting of a rotation matrix and a translation vector, is estimated and applied to update the source set of the point cloud. Finally, the algorithm evaluates whether the alignment error has converged or whether a predefined iteration limit has been reached. If convergence is not achieved, the above steps are repeated until the stopping criteria are satisfied, at which point, cloud alignment is completed.

Through this iterative process, the source set of the point cloud progressively approaches the geometric shape of the target set of point clouds, ultimately achieving good precision registration results.

**Figure 1:**
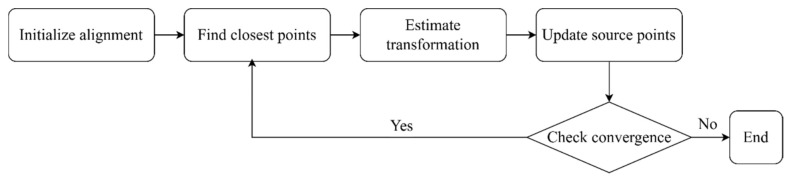
ICP flowchart.

As shown in Figure 1, this ICP flowchart illustrates a typical iterative procedure of point cloud registration. First, an initial alignment is applied to the source and target sets of the point cloud to establish a reasonable starting pose. Next, for each point in the source set of the point cloud, the nearest point in the target set of that point is searched to form point correspondences. After the correspondences are obtained, the system estimates the optimal rigid transformation, including rotation and translation, to minimize the geometric error between the points of matched pairs. The estimated transformation is then applied to the source set of the point cloud to update its pose. Finally, a convergence check is performed. If the convergence criterion is not satisfied, the algorithm returns to the nearest neighbor search and repeats the above steps. When the error is converged or a stopping condition is met, the process terminates to complete the alignment of the point cloud.

Let the source and target sets of point clouds be denoted as P and *Q*, respectively. Then, each point $p_{i}\in P$ and its corresponding point $q_{i}\in Q$ satisfy the same rigid transformation:

(6) $$q_{i}=Rp_{i}+t$$

where $R\in R^{3\times 3}$ is the 3D rotation matrix that represents the orientation difference between the two points, and $t\in R^{3}$ is the translation vector that describes their spatial displacement.

However, in practical applications, a perfect one-to-one correspondence between P and Q is rarely available. Therefore, the correspondences must be updated iteratively. Let

(7) $$C={\left\{(p_{i},\;q_{i})\right\}}_{i=1}^{n}$$

be the current set of matched point pairs, where n is the number of correspondences. The estimation of the optimal rigid transformation can then be formulated as minimizing the following sum of squared error objective:

(8) $$E\left(R,t\right)=\sum_{i=1}^{n}{\left[\delta_{i}^{T}(q_{i}-\left(Rp_{i}+t\right))\right]}^{2}$$

where $\left\|Rp_{i}+t-q_{i}\right\|$ denotes the Euclidean distance, and $\delta_{i}$ denotes the normal vector of surface associated with the target point $q_{i}$. Unlike the classical formulation of point-to-point ICP formulation, the algorithm of point-to-plane ICP minimizes the residual error projected onto the normal direction of local surface. Therefore, the optimization objective is to minimize the sum of squared point-to-plane distances between the transformed source points and the target surface.

Point-to-plane ICP incorporates local geometric information through the normal vectors of surface and generally provides faster convergence and improved registration accuracy when reliable normal vectors are available [33].

## 4. Numerical Examples

The previous section presented is only the theoretical foundations of point cloud stitching. In this study, the ICP algorithm based on point-to-plane is applied through repeated iterations to estimate 3D information as close as the actual object geometry. In addition, practical considerations are incorporated, and key parameters are tuned to improve computational efficiency. In this section, experiments show a structured light camera used to scan objects and construct their digital 3D representations. The experiments provide a detailed description of the point cloud stitching procedure, including comparisons of different methods and parameters.

**Figure 2:**
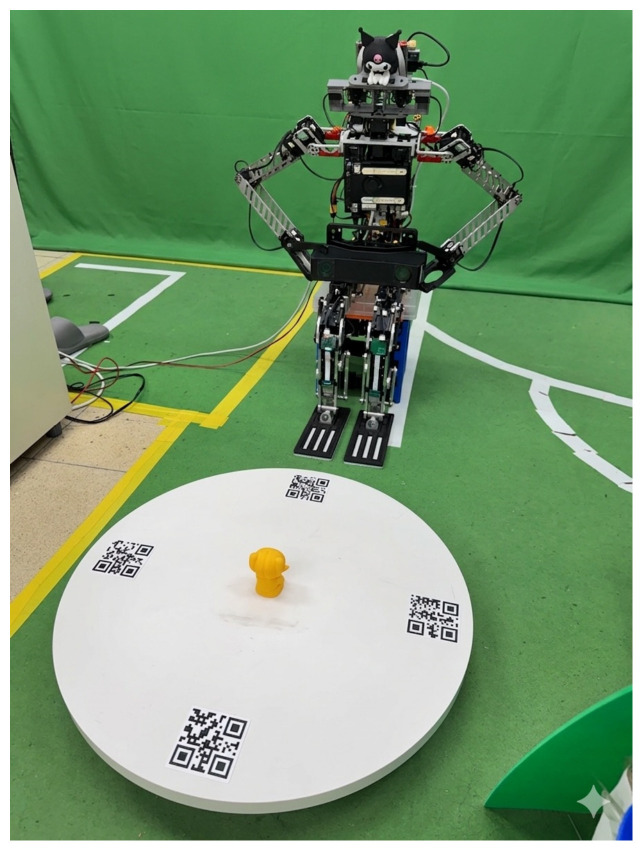
The humanoid holds the structured light camera and identifies the QR code on the rotating platform with its normal camera for the view angle of the object. The sets of point clouds captured by the structured light camera’s own view angles of the object used for the reconstruction of the 3D model.

The humanoid holds a structured light camera for point clouds of the object and identifies QR code on the rotary platform with its normal camera for the view angle as shown in Figure 2. To acquire the sets of point clouds from multiple viewpoints, the object is placed at the center of a rotary platform. Let the rotary platform rotate on a normal speed, and the structured light camera captures the object from different view angles identified by the camera on the humanoid. The system determines how many rotated degrees to store one set of point clouds; this can be set by the QR code recognized with the camera on the humanoid head. Thus, the sets of captured point clouds have their corresponding view angles for reconstructing a 3D model. A PC is connected to the structured light camera to collect the captured point cloud and to the humanoid for the view angles of the object. After collecting all data, the PC performs the stitching of the point cloud, and this results in the reconstructed 3D representation of the object.

**Figure 3:**
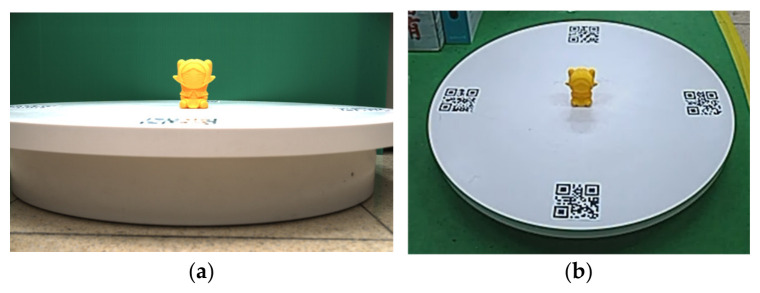
The pictures of object and platform: (**a**) image captured by the structured light camera, (**b**) image captured by humanoid camera.

Although the experimental study is of one object only, as shown in Figure 3, its shapes, including ears, face, head, legs, etc., are complex enough to be a representative object. In addition, because it is very sensitive to colors and surfaces, some tests are performed on the structured light camera before the experiments. The experiments show that the camera’s performance is not good for objects with red and green colors and for those with a metal surface. However, our structured light camera provides the function of adjusting light frequency. We just adjust the light frequency for yellow colors and plastic surfaces.

**Figure 4:**
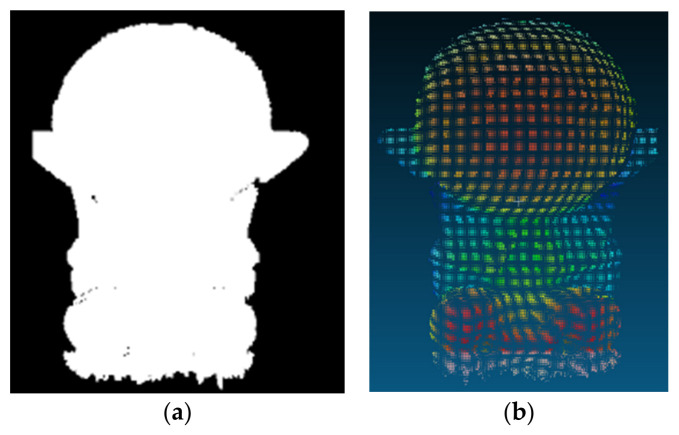
(**a**) Depth image and (**b**) colorized depth map captured by the structured light camera.

The object is placed at the center of a circular rotary platform whose height is approximately 11 cm above the ground. To capture the full surface contour and fine details of the object, the camera lens is aligned to be parallel to the target object. The humanoid system is set to store one set of point clouds when the platform rotates 5 degrees. And at each storage step, the system captures a 2D image, a depth map, and a 3D point cloud, as shown in Figure 3a and Figure 4, respectively. Since the humanoid camera is designed to identify the rotary angle of the platform, i.e., the view angle of structured light camera, the depth maps and point clouds consist of their corresponding angles for subsequent stitching and registration.

**Figure 5:**
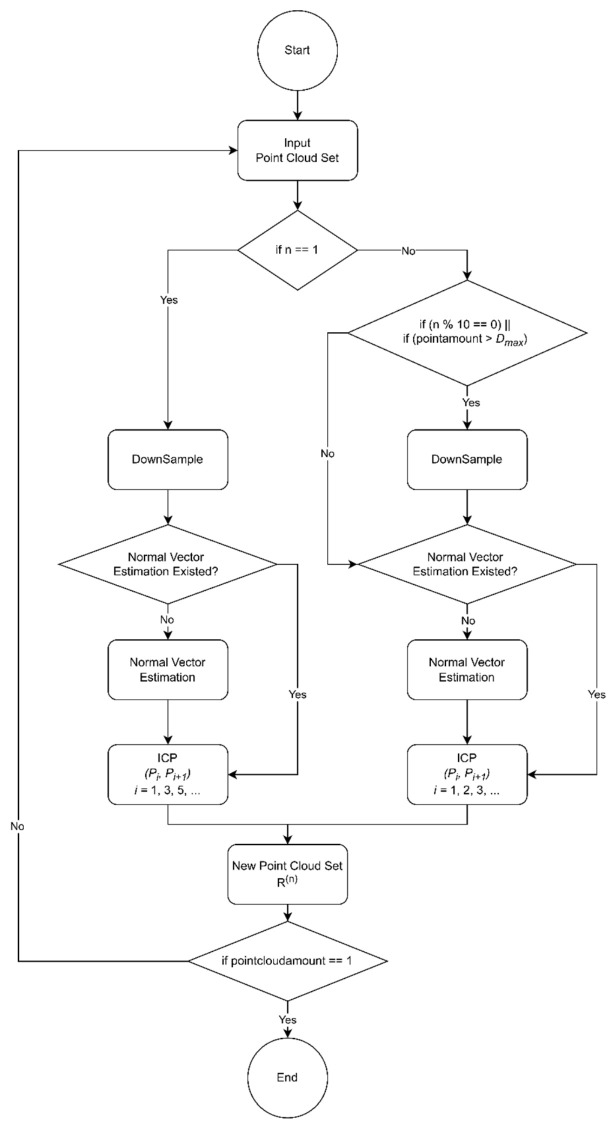
Flowchart of the proposed multi-view point cloud registration and merging pipeline.

After acquiring the point cloud of the object, multi-view point cloud stitching experiments are conducted by the block diagram of the stitching procedure as shown in Figure 5.

As illustrated in Figure 5, the proposed pipeline takes a multi-view point cloud set as input and iteratively performs preprocessing and pairwise registration to obtain a single merged point cloud. The process begins by checking whether the current iteration index n is 1. In the first iteration, the point cloud is downsampled to reduce data size and improve computational efficiency. The algorithm then verifies whether normal vectors are available. If normal information is absent, normal estimation is performed to provide the geometric attributes required for subsequent registration. ICP is then applied to register and merge the adjacent sets of point cloud—for example ${(P_{i},P}_{i+1})$ with i=1,3,5,… —resulting in an updated set of point clouds $R^{\left(n\right)}$.

For iterations where n≠1, the workflow includes an adaptive downsampling strategy to control the growth of the data volume. Downsampling has worked when either n mod 10= 0 or the total point count exceeds 500,000. Normal vectors are again checked and computed when necessary, after which ICP is performed to register and merge the pairs of point clouds sequentially—for example, i=1,2,3,… —producing a new group $R^{\left(n\right)}$. After each merging stage, the system evaluates whether the number of remaining point clouds has been reduced to one. If multiple point clouds remain, $R^{\left(n\right)}$ is fed back as the input for the next iteration and the procedure repeats until a single consolidated set of point clouds is obtained, completing the reconstruction process.

### 4.1. Implementation of Normal Vector Estimation

**Figure 6:**
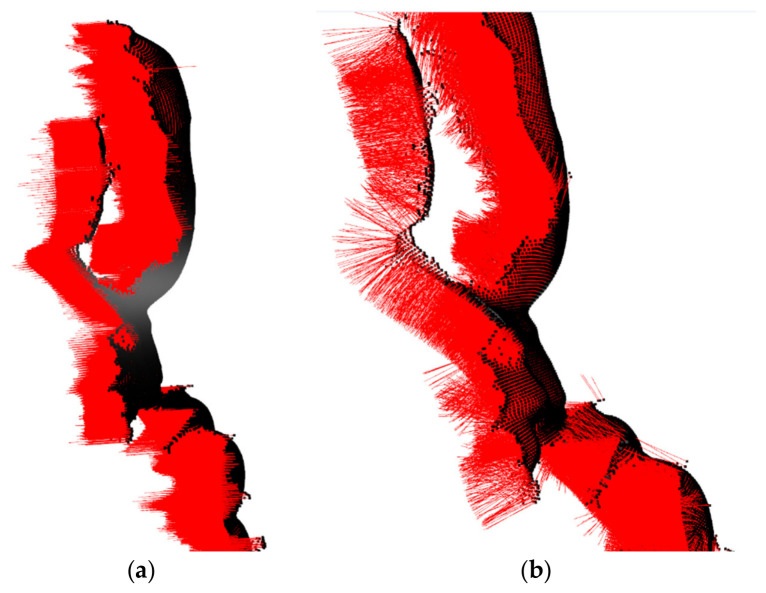
(**a**) Normal vector estimation results using Open3D. (**b**) The proposed custom implementation. The proposed method shows more pronounced normal direction changes at object bends on the right.

Since most of the files of point clouds captured by cameras do not include normal vector information, they must be obtained through estimation. During implementation, we observed the limitations when estimating normal vectors using Open3D. Therefore, this paper also develops an algorithm of normal vector estimation. In Figure 6, the left image shows the normal vector estimated using Open3D, while the right image shows the results produced by the proposed implementation. By comparing the two images in Figure 6, it can be clearly observed that at regions with bends or pronounced surface variations, the normal vectors estimated by the proposed custom implementation correctly reflect directional changes. Thus, the proposed improvement helps enhance the accuracy and stability of subsequent point cloud registration.

### 4.2. Implementation of Downsampling

**Figure 7:**
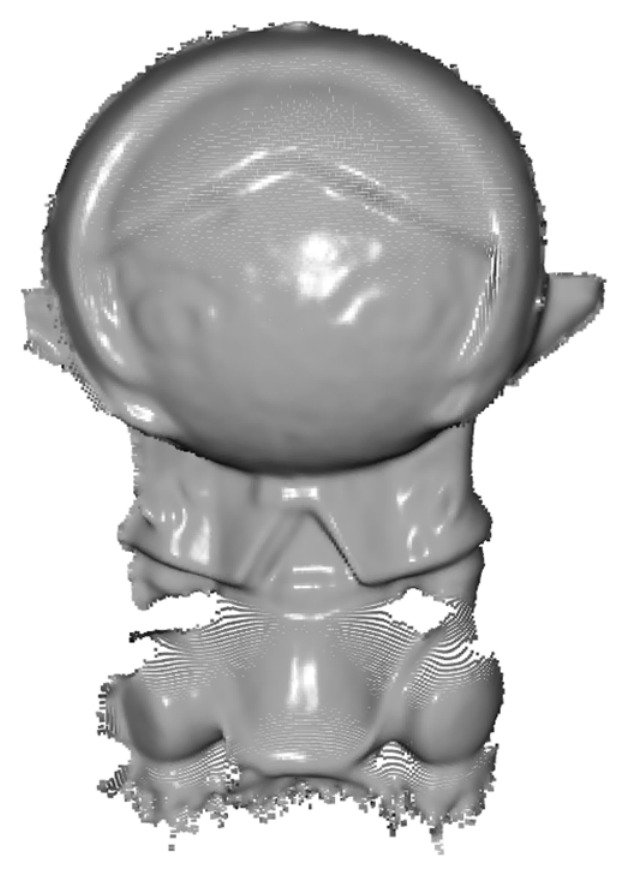
Source point cloud before downsampling.

**Figure 8:**
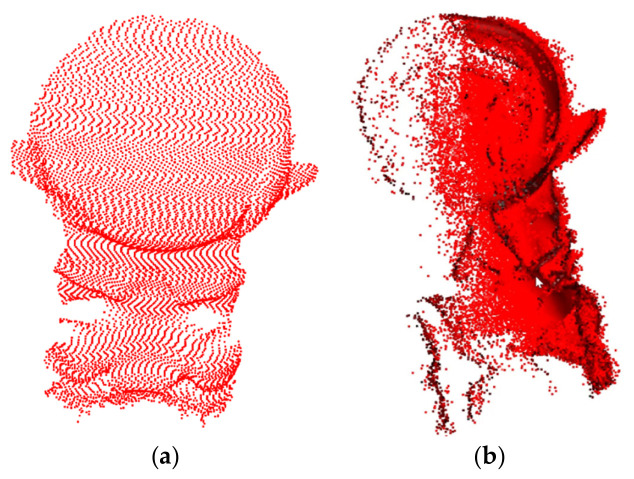
(**a**) Source point cloud after average downsampling. (**b**) Over-sparsification observed at the 38th round with average downsampling.

In the downsampling workflow, average downsampling was initially adopted. This method uniformly samples points across the dataset to reduce the point count and can improve overall processing efficiency, as shown in Table 1. However, average downsampling does not consider the spatial distribution of points, which leads to severe distortion and loss of geometric detail. In the experiments, starting from the point cloud of the original source as shown in Figure 7, we observed that after multiple rounds of average downsampling, even regions that had been successfully registered could be degraded due to excessive sparsification, as illustrated in Figure 8a,b. These results show that average downsampling is not a suitable strategy for this study.

**Figure 9:**
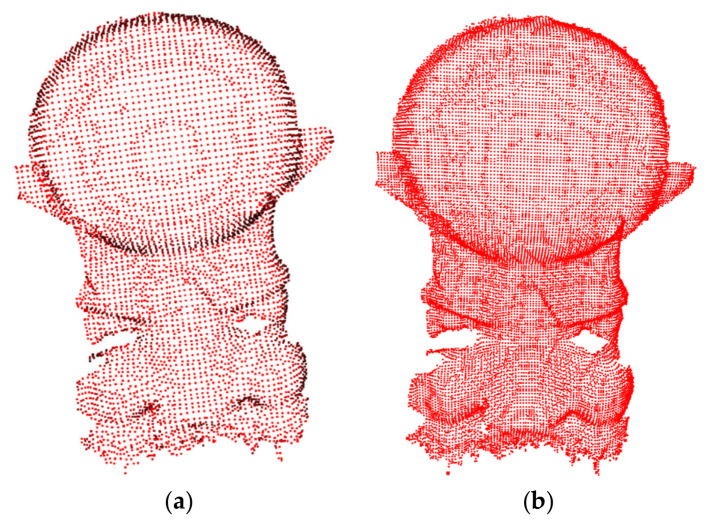
(**a**) Voxel downsampling result with voxel size set to 1. (**b**) Voxel downsampling result with voxel size set to 0.5.

**Figure 10:**
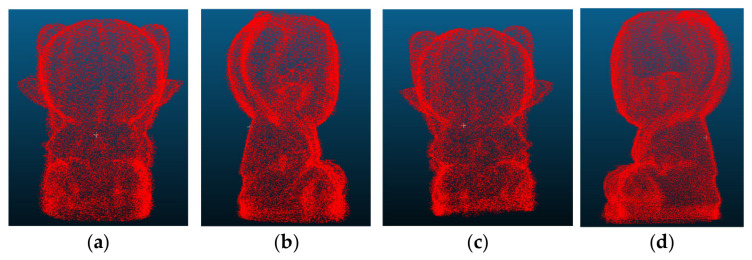
The stitching results, from left to right, are the front view (**a**), right-side view (**b**), rear view (**c**), and left-side view (**d**).

**Figure 11:**
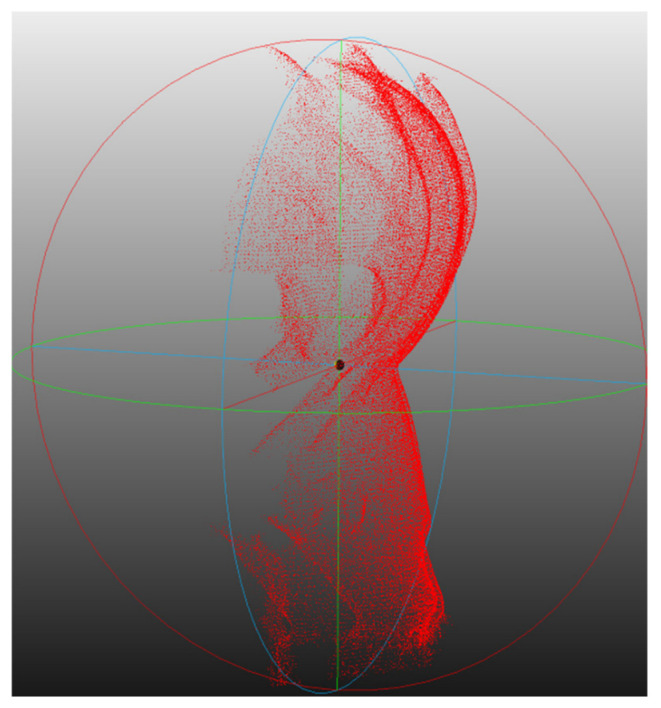
Registration failed on the back side of the object at the 18th round.

To compress a point cloud while preserving geometric details for computation efficiency, it is necessary to replace average downsampling with voxel downsampling. This method partitions the point cloud into voxels of a fixed size, and the points within each voxel are represented by their centroid as a representative point. This strategy preserves local spatial structures more effectively and reduces geometric distortion caused by excessive sparsification. As shown in Figure 9, an appropriate setting of the voxel size parameter allows the sampling density and reconstruction fidelity to be controlled, thereby retaining more point cloud information and improving reconstruction quality and detail recovery. As shown in Table 1, voxel downsampling requires more processing time than average downsampling. However, it produces a more complete 3D reconstruction than average downsampling, as demonstrated in Figure 8, Figure 9, Figure 10, Figure 11. Therefore, voxel downsampling is adopted as the downsampling method in this study.

### 4.3. The Optimization of Point Cloud Registration

In this study, stitching experiments using 76 sets of point clouds are conducted. First, all sets of point clouds are prepared for registration. At the beginning of the first iteration, a downsampling step is applied once, followed by checking whether each set of point clouds contains normal information. After preprocessing is completed, the workflow proceeds to point cloud registration and stitching.

Let the captured sequence sets of point clouds be denoted as:

(9) $$P=\{P_{1},\;P_{2},\;P_{3},\;\ldots ,\;P_{N}\}$$

where $P_{i}$ denotes the i -th set of point cloud, N=76 is the total number of point cloud sets, and the viewing angle between two consecutive sets of point clouds differs by 5 degrees.

Assuming that N is even, in each registration round, the system performs pairwise registration between two adjacent sets of point cloud, $P_{i}$ and $P_{i+1}$. Using the rigid transformation obtained from Equation (8), we get the registration function as follows:

(10) $$R_{(i+1)/2}^{\left(1\right)}=Reg\left(P_{i},\;P_{i+1}\right)\;,\;\;\;\;\;\;i=1,\;3,\;\ldots \;,\;(N-1)$$

Note that the output of registration function is the merged set of point clouds after alignment. In the first round, registration is performed in pairs as $P_{1}$ with $P_{2}$, $P_{3}$ with $P_{4}$, and so on. Therefore, after the pairwise registration step, the set numbers become N/2.

After completing the first registration round, the updated point cloud set based on $R_{(i+1)/2}^{\left(1\right)}$ in Equation (10) is given as follows:

(11) $$R_{(i+1)/2}^{\left(1\right)}=Reg\left(P_{i},\;P_{i+1}\right)\;,\;\;\;\;\;\;i=1,\;3,\;\ldots \;,\;(N-1)$$

In the next round, registration is performed in a sequential, overlapping manner rather than the non-overlapping pairing used in the first round. Specifically, $P_{1}^{\left(1\right)}$ is registered with $P_{2}^{\left(1\right)}$, then $P_{2}^{\left(1\right)}$ is registered with $P_{3}^{\left(1\right)}$, and so on. Following this process, the second round of registration becomes as follows:

(12) $$R_{j}^{\left(m+1\right)}=Reg\left(P_{j}^{\left(m\right)},\;P_{j+1}^{\left(m\right)}\right),\;\;m=2,\;3,\;\ldots ,\;M\;\mathrm{and}\;j=1,\;\ldots ,\;M\;-\;1$$

where M=N/2−(m−1) because under sequential overlapping registration, each round reduces the set numbers of the point cloud by one. This procedure forms a binary merge tree structure, where the set numbers of the point cloud are decreased at each round until only a single final result remains:

(13) $$P_{final}=P_{1}^{\left(N^{*}\right)}$$

where $N^{*}$ denotes the final iteration index, and $P_{\mathrm{final}}$ is the final stitched 3D result. After each registration round, the output is stored in the point cloud set labeled with the current iteration number. The iteration counter is then incremented to start the next registration round.

At the beginning of each new round, the system first checks whether the current iteration satisfies the downsampling condition. If the condition is met, downsampling is performed; otherwise, this step is skipped. Because the information of a normal vector may be lost during preprocessing, the system then verifies whether the current set of point clouds still contains normal vectors. If the normal vectors are available, its computation is skipped; if not, normal vectors are re-estimated. Once these prerequisites are satisfied, a new round of point cloud registration is initiated.

The stitching results are presented in Figure 10, including the front view, right-side view, rear view, and left-side view. In addition, to improve registration efficiency, this study further investigates the effect of different initial merging strategies. Before stitching, pairwise preregistration is applied to merge the original 76 sets of point clouds into 38 updated sets of point clouds.

To quantitatively evaluate the geometric accuracy of the stitched point cloud, the reconstructed point cloud, $P_{final}$, is further compared with the reference point cloud of the 3D model of the target object. Actually, the 3D model of the target object is the STL model for a 3D printer. During the verification phase, the STL model generated by CAD is first imported into Open3D, and uniform sampling is performed on the model surface to generate a reference point cloud. Since the STL model and the structured light reconstructed point cloud are located in different coordinate systems, rigid registration is required first. Next, the geometric centers of the two models are used for coarse alignment to ensure that the two models have a similar positional relationship. Then, the iterative closest point (ICP) algorithm is used for fine registration. By repeatedly searching for the nearest point and minimizing the point-to-point distance error, the optimal rotation matrix and translation vector are obtained to establish a common coordinate system between the STL model and the reconstructed point cloud. After registration is completed, the STL-sampled point cloud is used as the reference model. The nearest neighbor distance between the reconstructed point cloud and the reference model is calculated.

The system performance of the registration is compared with G-ICP as well. Several common metrics are engaged for the quantitative evaluation, including root mean square rrror (RMSE), mean error, median error, Chamfer distance, and Hausdorff distance. Note that Chamfer distance evaluates the bidirectional geometric similarity between the reconstructed model and the reference model, while Hausdorff distance reflects the maximum deviation between the two datasets and indicates the worst-case geometric error. These metrics quantify the geometric deviation between the models of reconstructed and reference from different perspectives, including average error, bidirectional similarity, and maximum deviation. Eventually, the comparing study tries to include Open 3D and FGR. But both cannot conduct the registration of the object. Thus, they cannot be evaluated by system performance. The comparison results of quantitative evaluation are summarized in Table 2. As shown in Table 2, by the proposed ICP, the stitching framework of the proposed multi-view point cloud can reconstruct the geometric structure of the target object with good accuracy and consistency. And the reconstructed model of the proposed ICP is statistically equivalent to that of G-ICP in local registration accuracy, while the proposed method is faster and more robust to measurement noise; its higher cumulative rotational drift is characterized and mitigated by pose-graph optimization in the added evaluation on Section 4.4.

During the stitching experiments of the multi-view point cloud, processing large-scale data of the point cloud increases computation time. To improve registration efficiency, voxel downsampling is performed at specific registration rounds or when the point cloud size exceeds a predefined threshold. This reduces subsequent computation while maintaining registration stability. The threshold design follows two principles. First, an upper limit is imposed on the accumulated count of points. When the merged point cloud exceeds the threshold $D_{max}$ —for example, $D_{max}=5\times 10^{5}$ points—the system automatically triggers a downsampling step to prevent memory overflow or excessive computation latency caused by overly large data. Second, a round-based control rule is applied. After a fixed number of pairwise merge rounds, for example, every five rounds, downsampling is executed. This dual threshold mechanism controls point cloud density and ensures a balance between efficiency and accuracy throughout the stitching process.

To evaluate stitching efficiency, we tested a strategy in which downsampling is always triggered when the point count exceeds 500,000, while the variable parameter is performing an additional downsampling step every K registration rounds. The first comparison measured the runtime for stitching the original 76 sets of point clouds. The runtime results of K from 5 to 15 are summarized in Table 3. As shown in Table 3, increasing K can decrease computation time while still maintaining a complete reconstruction.

Based on the above experiments, performing pairwise preregistration before stitching reduces the set numbers of the point cloud and improves overall efficiency. The results are summarized in Table 4. This experiment also compares the impact of applying voxel downsampling once every five rounds versus once every ten rounds during the stitching process. The results show that performing voxel downsampling once every ten rounds saves 12 min, corresponding to a 25% reduction in total runtime. This result is used as a reference criterion for deciding when to apply downsampling in the registration pipeline described above.

A three-at-a-time merging strategy is also evaluated to compare how different merging schemes influence registration efficiency, thereby optimizing the overall stitching pipeline. If three sets of point clouds are preregistered as a group, the total set numbers of the point cloud can be reduced to 25. However, the final stitching result fails clearly. As shown in Figure 11, the back side of the object contains fewer distinctive features, and the difference between the two sets of point clouds is relatively large, causing both sets to misalign and fail to overlap properly. This test indicates that three-sets-at-a time preregistration is not an appropriate strategy.

The computational complexity of the proposed ICP in this study is analyzed as follows. The processes of point cloud registration include three steps: voxel downsampling, normal estimation, and iterative closest point registration (ICP). Voxel downsampling has a time complexity of O(N), and normal estimation uses a k-d tree for neighborhood search, with a complexity of O(NlogN). ICP registration requires nearest neighbor search and rigid body transformation estimation in each iteration, with a time complexity of approximately O(I⋅NlogN), where I is the number of ICP iterations.

The proposed ICP is conducted by statistical validation too. Basically, the pipeline is deterministic, so variability is quantified over the 76 pairwise registrations and through controlled randomized trials. The 95% confidence interval (CI) of the mean is computed with the Student’s t-distribution as follows:

(14) $$95\%\;CI\;=\;mean\;\pm \;t_{0.975,\;N-1}\frac{s}{\sqrt{N}}$$

where s is standard deviation, N is the numbers of point cloud sets (the parameter of the Student’s t-distribution). The reproducibility of the experiments is evaluated by (14).

**Figure 12:**
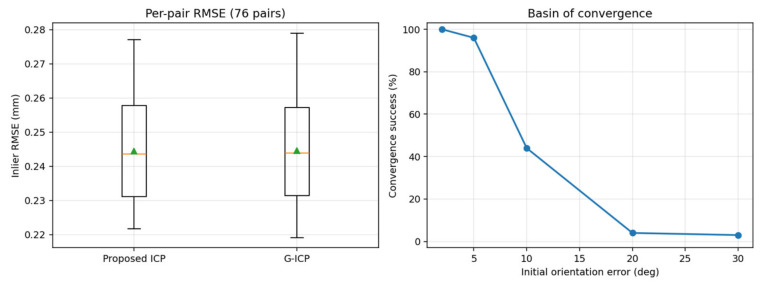
Distribution of per-pair inlier RMSE (**left**) and convergence success rate versus initial orientation error (**right**).

Note that because the standard deviation of 76 pairs is hard to define and because we cannot utilize that of normal distribution, Student’s t-distribution is applied in this experiment. Over the 76 pairwise registrations, the mean inlier RMSE of the proposed ICP and G-ICP is 0.2444 ± 0.0151 mm and 0.2446 ± 0.0154 mm, respectively, as shown in Table 5 and Figure 12 (left). The paired Wilcoxon signed-rank test and the paired t-test both yield *p* = 0.12 (no significant difference). At the initial orientation error ±5°, the basin-of-convergence analysis according to (14) further shows a 96% success rate by the proposed ICP and G-ICP as illustrated in Figure 12 (right). As for the initial orientation error at ±10°, the success rate drops sharply beyond this point. These results confirm that the 5° angular increment or decrement used during acquisition lies safely within the reliable convergence range. Consequently, the proposed ICP and G-ICP show no significant difference in statistical validation.

Robustness to noise and outliers are evaluated as follows. Synthetic perturbations are injected into the measured clouds, using each method’s clean solution as the reference pose. The surface registration error is the RMS displacement of the object surface under the estimated pose.

(15) $$e_{surf}=\sqrt{\frac{1}{n}\;\sum_{i}{\left\|T_{ref}p_{i}-T_{est}p_{i}\right\|}^{2}}$$

where n is the number of points in a set of point cloud, and $T_{ref}$ and $T_{est}$ are the transformation with reference and estimation, respectively.

**Figure 13:**
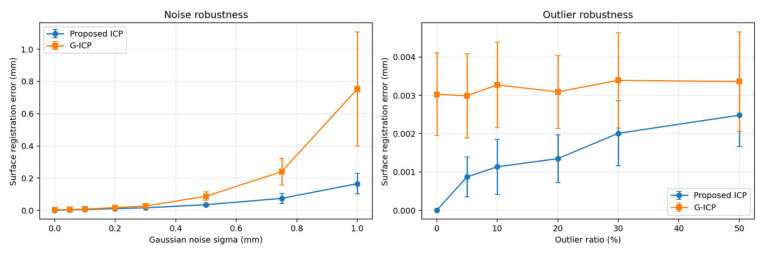
Surface registration error versus Gaussian-noise level (**left**) and outlier ratio (**right**).

Under zero-mean Gaussian noise and a uniform outlier, the proposed ICP is compared with G-ICP at many levels as shown in Table 6 and Figure 13. In Table 6, the comparison of noise levels is from 0.00 to 1.00, and that of outlier is from 0% to 50%. Under Gaussian noise σ = 1.00 mm, the surface registrations keep the error in mean values at 0.1655 and 0.7530 with the proposed ICP and G-ICP, respectively. This result shows that the proposed ICP is better at eliminating the influence of noise disturbance. Because the size of voxel downsampling is set at the threshold of 0.75 mm, the outliers greater than the threshold are rejected by the proposed ICP. As shown in Table 6, for the outlier, the proposed ICP has excellent rejection. As a result, for noise elimination and outlier rejection, the proposed ICP is better than G-ICP.

### 4.4. The Evaluation of Global Consistency and Cumulative Drift

In this subsection, global consistency and cumulative drift are evaluated. All results are computed on the full sets of 76 structured light point clouds using the preprocessing pipeline with the voxel downsampling at ν = 0.5 mm (edge length) and K = 15 (nearest neighbor points) for normal estimation. With a correspondence distance threshold of 0.75 mm and identity initialization, the global accuracy is additionally evaluated against the CAD (STL) reference in this subsection too.

Because the acquisition covers 76 views at 5° (5° × 76 = 380°), it forms a closed loop. Global consistency is evaluated through the loop-closure residual of the chained pairwise transformations: composing the 75 sequential rigid transforms with the wrap-around registration T_wrap_ should equal the identity, and its deviation is the accumulated drift:

(16) $$L\;=\;T_{wrap}\;\cdot \;T_{n-2}\;\cdots \;T_{1}\;\cdot \;T_{0}$$

(17) $$\theta_{drift}=arccos\left(\frac{1}{2}\left(tr\;R_{L}-1\right)\right)\;,\;d_{drift}=\|t_{L}\|$$

**Figure 14:**
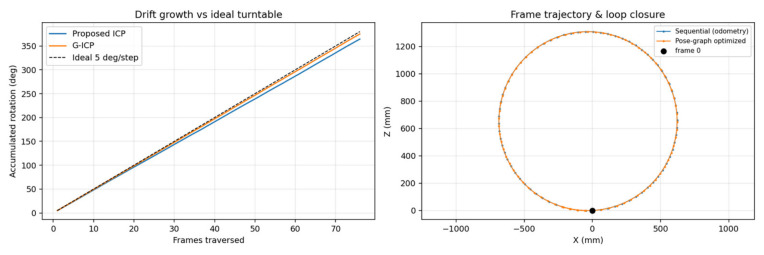
Accumulated rotation versus the ideal 5°/step (**left**) and the camera-frame trajectory before and after pose-graph optimization (**right**).

The proposed ICP and G-ICP are compared by these performance metrics in Table 7. Both are locally uniform and accurate, but the proposed point-to-plane ICP accumulates a larger loop-closure drift (14.9°/170.5 mm) than G-ICP (4.3°/48.7 mm), caused by a small systematic per-step under-rotation (4.79° vs. 4.93° per 5° step), as shown in Figure 14, where the left panel plots the accumulated rotation against the ideal 5°/step and the right panel shows the camera-frame trajectory before and after pose-graph optimization.

**Figure 15:**
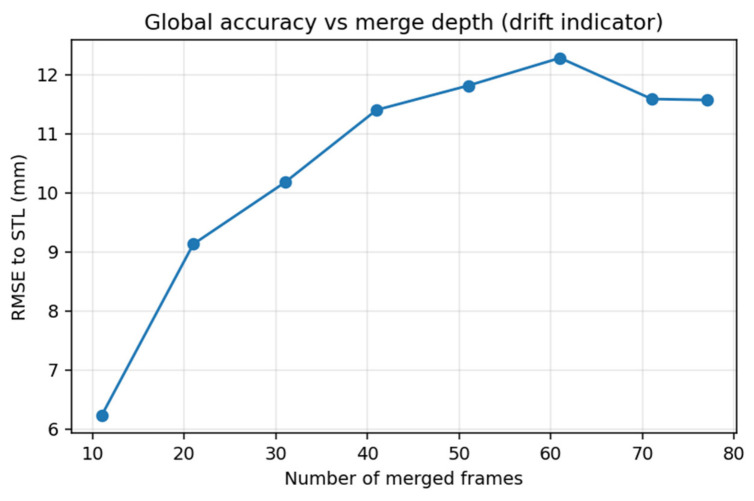
Nearest neighbor RMSE of the sequentially merged reconstruction to the CAD reference versus the number of merged views (drift indicator).

As direct ground-truth evidence, the sequentially merged reconstruction was compared with the CAD model at increasing merge depths, using the same nearest neighbor distance and centroid-plus-global-ICP alignment as Table 2. The reconstruction-to-CAD RMSE grows from about 6.2 mm at 11 views to a peak of about 12.3 mm as drift accumulates and then settles near 11.6 mm once the 380° sequence closes the loop as shown in Figure 15. This trend is consistent with the large Chamfer (47.2 mm) and Hausdorff (80.9 mm) distances already reported in Table 2.

In this section, the experiments demonstrate that the ICP registration algorithm is an effective approach for a 3D object stitched by the sets of point clouds. However, there remains substantial room to improve computational efficiency. In the early stage of the experiments, when efficiency was not considered, the stitching process required up to 48 h, approximately two days. After a detailed investigation of computational efficiency, the key points can be summarized as follows:

• Efficiently reducing the set numbers of the point cloud is critical.
• Downsampling reduces the number of points in each set of point clouds. Average downsampling leads to substantial loss of 3D information and is therefore not feasible.
• Downsampling must not lose the information of the normal vector; otherwise, the normal vector must be estimated again.

## 5. Conclusions and Future Works

In this paper, a humanoid capturing the sets of point clouds and their view angles by a structured light camera and normal camera, respectively, to reconstruct a 3D digital model is proposed. In addition, the stitching method of point clouds with multi-view angles based on the ICP registration algorithm is proposed. In particular, the structured light camera is engaged to achieve automatic stitching without relying on manual calibration or positioning. During data acquisition, the rotation angle of the platform is identified to ensure that the sets of point clouds captured from adjacent viewpoints contain sufficiently large overlapping regions. This design improves the stability and accuracy of the iterative ICP registration process. Experimental results demonstrate that point cloud stitching can be achieved even without manual alignment in the early stage. The results indicate that the proposed method is efficient and highly automated.

In this paper, the stitching problem of point clouds with the objective of reconstructing a complete 3D model of the target object is addressed. To achieve this goal, appropriate point cloud preprocessing strategies are incorporated. In particular, normal vector estimation and downsampling are designed and selected to optimize both runtime and reconstruction quality.

Beyond the experimental validation, this study also provides a systematic analysis of the influence of preprocessing operations on the stability of ICP-based point cloud registration. The results demonstrate that appropriate combinations of downsampling strategies and normal vector estimation can effectively improve both the robustness and computational efficiency of multi-view point cloud stitching. This finding provides useful insights for designing efficient preprocessing pipelines for ICP-based 3D reconstruction systems.

In the final stitching experiments, 3D point cloud reconstruction with high quality was achieved successfully. Voxel downsampling can remove stored normal vectors. Thus, the normal vectors of the surface must be estimated again to maintain stitching accuracy after each downsampling step.

To balance the control and runtime of point cloud density, we first verified that the proposed pipeline can produce high-quality results using the original 76 sets of point clouds. We then further reduced the initial set numbers of point clouds to decrease the required registration rounds and shorten the overall computation time. In parallel, a fixed round downsampling strategy was evaluated to effectively control the growth and processing time of the point cloud. Experimental results show that reducing the input set numbers of point clouds can significantly improve computational speed, and reducing the frequency of fixed schedule downsampling can further enhance efficiency.

Overall, the experimental results confirm that accurate multi-view point cloud stitching can be achieved without manual calibration. However, the performance of this approach depends more strongly on the quality of point cloud preprocessing to ensure data completeness and accuracy. In addition, the viewpoint difference between successive scans should not be too large; otherwise, registration failures are more likely to occur.

In the comparison study, the proposed ICP is better at the quantitative evaluation of system performance and the rejection of noise and outliers. In the comparison of global consistency and cumulative drift, the proposed ICP is unfortunately worse than G-ICP on loop-closure rotation and translation residuals. This comparison suggests that one should study the improvement in the proposed ICP for cumulative drift on a loop-closure residual.

This study aims to make the reconstruction of an object’s 3D information less constrained by environmental conditions, object placement, and camera positioning. However, because no external positioning assistance is used, if uniform and stable rotation of the object during acquisition cannot be ensured, registration may still fail even with a well-designed preprocessing pipeline. This is mainly because excessive differences between adjacent sets of point clouds can reduce overlap and correspondence reliability.

To address this limitation, future work can focus on improving the spatial similarity between the source and target sets of point clouds prior to registration, thereby increasing the registration success rate. Some advanced preprocessing methods, such as adaptive neighborhood selection, outlier rejection, or feature-preserving downsampling, can reduce computational time or promote performance. They are interesting research topics for the future.

To enable a more flexible acquisition setup, a non-contact sensor can be integrated on the camera side, such as an IMU (inertial measurement unit). This approach is suitable for scenarios where the object remains stationary while the camera moves. The IMU can provide estimates of the camera motion and orientation changes in 3D space, which can be used to obtain an initial spatial alignment between two sets of point clouds before registration. As a result, the robustness and accuracy of automatic registration can be achieved.

Furthermore, the proposed framework provides a practical reference for future research on automated 3D reconstruction systems, especially in applications such as industrial inspection, digital archiving, and robotic perception. The integration of sensor-based motion estimation and adaptive preprocessing strategies may further improve the robustness of multi-view point cloud registration under more complex acquisition environments. In addition, humanoids are a high mobility platform. It is possible to let the humanoid take a structured light camera to move around the object for point clouds with multi-view angles. Then, the humanoid can reconstruct a 3D digital model easier and faster. However, it is a great challenge and an interesting research topic to localize the posture of the humanoid.

## Figures and Tables

**Table 1:** Runtime of downsampling methods.

Method	Time (Second)
Voxel downsampling using Open3D	0.0022 s
Average downsampling	0.0001 s
Voxel downsampling	0.2682 s

**Table 2:** The comparison of quantitative evaluation between proposed ICP and G-ICP.

Metric	Proposed ICP	G-ICP	Better
RMSE	0.239	0.256	Proposed ICP
Mean error	0.222	0.238	Proposed ICP
Median error	0.221	0.237	Proposed ICP
Std Dev (mm)	0.089	0.093	Proposed ICP
Max. error	0.483	0.466	G-ICP
P95 error (mm)	0.363	0.387	Proposed ICP
Chamfer distance	47.239	47.216	Almost same
Hausdorff distance	80.866	80.860	Almost same
Computation time	3 h 17 min	3 h 30 min	Proposed ICP

**Table 3:** Runtime for stitching 76 point clouds after 76 registration rounds.

K	5	10	15
Start time	23:19	01:24	01:35
End time	01:57	03:55	04:05
Total time	3 h 36 min	3 h 30 min	3 h 20 min

**Table 4:** The 38 sets of point clouds are stitched. The runtimes obtained using downsampling once every five rounds and once every ten rounds are summarized in the table, based on 37 registration rounds.

	Once Every 5 Rounds	Once Every 10 Rounds
Start time	23:43	01:24
End time	00:31	02:00
Total time	48 min	36 min

**Table 5:** Per-pair registration statistics over the 76 pairs.

Metric (Over 76 Pairs)	Proposed ICP	G-ICP
Inlier RMSE, mean ± SD (mm)	0.2444 ± 0.0151	0.2446 ± 0.0154
95% CI of the mean (mm)	[0.2410, 0.2479]	[0.2411, 0.2481]
Paired difference	Wilcoxon *p* = 0.12 (n.s.)	

**Table 6:** Surface registration error (mm) under noise and outliers.

Condition	Proposed ICP (mm)	G-ICP (mm)
Gaussian noise σ = 0.00 mm	0.0000 ± 0.0000	0.0030 ± 0.0011
Gaussian noise σ = 0.05 mm	0.0027 ± 0.0009	0.0044 ± 0.0018
Gaussian noise σ = 0.10 mm	0.0048 ± 0.0011	0.0075 ± 0.0032
Gaussian noise σ = 0.20 mm	0.0109 ± 0.0037	0.0171 ± 0.0068
Gaussian noise σ = 0.30 mm	0.0155 ± 0.0042	0.0265 ± 0.0090
Gaussian noise σ = 0.50 mm	0.0347 ± 0.0102	0.0873 ± 0.0267
Gaussian noise σ = 0.75 mm	0.0742 ± 0.0313	0.2399 ± 0.0831
Gaussian noise σ = 1.00 mm	0.1654 ± 0.0631	0.7529 ± 0.3551
Uniform outliers, 0%	0.0000 ± 0.0000	0.0030 ± 0.0011
Uniform outliers, 5%	0.0009 ± 0.0005	0.0030 ± 0.0011
Uniform outliers, 10%	0.0011 ± 0.0007	0.0033 ± 0.0011
Uniform outliers, 20%	0.0013 ± 0.0006	0.0031 ± 0.0010
Uniform outliers, 30%	0.0020 ± 0.0008	0.0034 ± 0.0012
Uniform outliers, 50%	0.0025 ± 0.0008	0.0034 ± 0.0013

**Table 7:** Global-consistency and per-pair metrics over the full loop: proposed ICP versus G-ICP.

Metric	Proposed ICP	G-ICP
Mean rotation per 5° step (°)	4.79 ± 0.11	4.93 ± 0.09
Loop-closure rotation residual (°)	14.9	4.3
Loop-closure translation residual (mm)	170.5	48.7
Per-pair fitness	0.962 ± 0.017	0.961 ± 0.017
Per-pair inlier RMSE (mm)	0.2444 ± 0.0151	0.2446 ± 0.0154

## Data Availability

Data is unavailable due to privacy restrict.
